# Technical efficiency of public district hospitals in Madhya Pradesh, India: a data envelopment analysis

**DOI:** 10.3402/gha.v6i0.21742

**Published:** 2013-09-24

**Authors:** Tej Ram Jat, Miguel San Sebastian

**Affiliations:** 1 United Nations Population Fund, Bhopal, India; 2 Department of Public Health and Clinical Medicine, Epidemiology and Global Health, Umeå University, Umeå, Sweden; 3 Swedish Research School for Global Health, Umeå University, Umeå, Sweden

**Keywords:** health services, hospital efficiency, data envelopment analysis, Madhya Pradesh, India

## Abstract

**Background:**

Scarcity of resources for healthcare is a well-acknowledged problem. In this context, efficient utilization of existing financial and human resources becomes crucial for strengthening the healthcare delivery. The assessment of efficiency of health facilities can guide decision makers in ensuring the optimum utilization of available resources.

**Objective:**

The objective of this study was to evaluate the technical efficiency (TE) of the public district hospitals in Madhya Pradesh, India, with special emphasis on maternal healthcare services, using data envelopment analysis (DEA).

**Methods:**

Data from 40 district hospitals from January to December 2010 were collected from the health management information system and other records of the department of health and family welfare of the state. DEA was performed with input orientation and variable returns to scale assumption.

**Results:**

TE and scale efficiency scores of the district hospitals were 0.90 (SD=0.14) and 0.88 (SD=0.15), respectively. Of the total district hospitals in the study, 20 (50%) were technically efficient constituting the ‘best practice frontier’. The other half were technically inefficient, with an average TE score of 0.79 (SD=0.12) meaning that these hospitals could produce the same outputs by using 21% less inputs from current input levels. Twenty-six (65%) district hospitals were found to be scale inefficient, manifesting a mean score of 0.81 (SD=0.16).

**Conclusions:**

Half of the district hospitals in the study were operating inefficiently. Decision makers and administrators in the state should identify the causes of the observed inefficiencies and take appropriate measures to increase efficiency of these hospitals.

Scarcity of resources for healthcare is a well-acknowledged problem. The public sector of healthcare in India is facing the constraints of financial resources as well as a shortage of health professionals at all levels (1). In this context, the efficient utilization of existing financial and human resources becomes crucial for strengthening the healthcare delivery in the country. The assessment of efficiency of health facilities can guide decision makers in ensuring the optimum utilization of the available resources.

Data envelopment analysis (DEA) has emerged as an effective and popular method for evaluating the efficiency of decision-making units (DMUs) in different sectors including the health sector. There have been a number of studies on assessing the efficiency of hospitals, health centers and the overall healthcare system by using DEA in different settings. These studies have been conducted in industrialized countries as well as in middle- and low-income countries (2–16), including India (17–20). Some researchers have also undertaken extensive reviews of efficiency studies using DEA in the healthcare sector (21–24). However, thus far there is no research available on the efficiency assessment of hospitals in Madhya Pradesh, India.

Geographically, this state is the second largest in the country and it consists of around 6% Indian population. Madhya Pradesh suffers 269 maternal deaths for every 100,000 live births and is among the states with the highest maternal mortality ratio (MMR) in the country (25). The state aimed at achieving an ambitious target of reducing MMR to 220 per 100,000 live births by the year 2012, which could not be achieved. Now the state has set up a target to achieve this by the year 2017 (26). To achieve this, the state government is making concentrated efforts to increase the availability and use of maternal healthcare services including institutional deliveries and emergency obstetric care services. As part of these efforts, district hospitals are being strengthened as comprehensive emergency obstetric care centers. District hospitals are key resource units in the healthcare system of the state and they consume a major share of resources for healthcare. Efficient district hospitals can contribute considerably toward achieving the reduction in MMR. Therefore, capturing and monitoring their inefficiencies has become critical.

In this context, the objective of this study was to evaluate the technical efficiency (TE) of the public district hospitals in Madhya Pradesh, with special emphasis on maternal healthcare services, using DEA. The results of this study will be useful for the decision makers and administrators in reviewing and taking appropriate measures for improving the performance of these hospitals.

## Methods

### Study area

Madhya Pradesh is situated in the central part of India. As per the provisional figures of the 2011 census, the state has a population of 72,597,565. Population density in the state is 236 person per square kilometer (27). The state comprises 55,393 villages, 313 development blocks, and 50 administrative districts. Table 1 presents the comparative status of key demographic and socio-economic indicators of Madhya Pradesh and India, which shows that the state has poorer health indicators in comparison with the country as a whole (25, 27, 28).

**Table 1:** Key demographic and socio-economic indicators of Madhya Pradesh state and India

S. no.	Indicator	Madhya Pradesh	India
1.	Total population (Census 2011)	72,597,565	1,210,193,422
	1.1 Females (%)	48.19	48.46
	1.2 Males (%)	51.81	51.54
2.	Density of population (Per sq. km.)	236	382
3.	Decadal population growth 2001–2011 (%)	20.30	17.64
4.	Total literacy (%) (2011)	70.60	74.04
	4.1 Female literacy (%)	60.00	65.46
	4.2 Male literacy (%)	80.50	82.14
5.	Families living below poverty line (%)	38	40
6.	Maternal mortality ratio (2007–09)	269	212
7.	Infant mortality rate (2007–09)	67	50

The health system in the state consists of a three-tier structure having primary, secondary, and tertiary healthcare facilities. The primary tier has three types of facilities; (1) a Sub Health Centre (SHC) for every 3,000–5,000 population, (2) a Primary Health Centre (PHC) for every 20,000–30,000 population, and (3) a Community Health Centre (CHC) to serve as a referral center for PHCs in its jurisdiction covering a population of 80,000–120,000. The secondary tier consists of district hospitals and civil hospitals (sub-district hospitals), which provide secondary-level referral and specialist services along with providing primary care services for urban areas. Tertiary-level healthcare is provided by medical colleges and apex institutions (29).

The responsibilities of delivering healthcare services in the state lie with the state government. As of March 2010, there were 5 medical colleges, 50 district hospitals, 56 civil hospitals, 333 CHC, 1,155 PHC, and 8,869 SHC functioning in the public sector in the state (30, 31). The state also has a huge network of private-sector healthcare facilities, most of which are situated in urban areas.

### DEA conceptual framework

Two frontier methodologies, stochastic frontier analysis (SFA) and DEA are commonly used for measuring efficiency of healthcare organizations (24). SFA is a parametric approach that uses econometric techniques to estimate efficiency of DMUs. It constructs a smooth parametric frontier and allows for the possibility of modeling and measurement error. SFA appeals to economic theory when considering the shape of the frontier and the statistical criteria that might be used to differentiate the appropriateness of alternative functional relationships for particular data sets (32). As Jacobs et al. mention, advocates of DEA would argue that the problems of providing a prior specification of functional form can be avoided by applying a non-parametric technique. Consequently, DEA is highly flexible, the frontier moulding itself to the data (33). DEA has been recommended for evaluating the hospital efficiency in settings with inefficient health-sector information and particularly inappropriate data availability on prices of inputs (6, 8, 34). It was essential in this study to use an approach suitable for measuring the efficiency of hospitals that use multiple inputs to produce multiple outputs. In contrast to parametric methods such as SFA, the non-parametric properties of DEA provide that required flexibility (33). DEA is a non-parametric mathematical programming approach to frontier estimation, which was first developed by Charnes et al. (35) to measure efficiency of production units with multiple inputs and outputs and it was extended by Banker et al. (36). They developed DEA models building upon the work of Farrell (37). DEA uses linear programming techniques to evaluate the relative efficiency of each DMU, for example, district hospitals, health centers, nursing homes, and so on. It constructs production frontiers and measures the efficiency of a DMU relative to these constructed frontiers by using a mathematical programming technique. It is used to evaluate relative performance in a group of DMUs in which all members are fairly homogenous and use an identical set of inputs to produce a variety of identical outputs. It means that the yardstick for comparing the efficiency of a particular DMU is determined by the group of DMUs included in the study sample. The efficient DMUs that compose the ‘best practice frontier’ are assigned an efficiency score of 1 (or 100%) and are considered technically efficient in comparison with their peers. The inefficient DMUs are assigned a score between 1 and 0 (38).

DEA, as an analysis tool, has flexibility in handling multiple inputs and outputs, which makes it suitable for measuring the efficiency of hospitals that use multiple inputs to produce multiple outputs. However, it produces results, which are sensitive to measurement error, and it measures the efficiency relative to the best practicing DMUs within the sample of DMUs included in the study. Thus, it does not allow the comparison of the TE with DMUs outside the sample (39). Another shortcoming of DEA is that it captures the best among the sample but we do not know if these best DMUs can perform better. This is because DEA estimates the relative efficiency of a DMU compared to its peers but not the absolute efficiency such as a theoretical maximum efficiency of a DMU.

DEA results can be used by the decision makers and administrators as inputs in making informed decisions regarding the planning, allocation, and utilization of resources. The information generated by DEA on output inefficiencies and excess inputs can be substantially utilized for the monitoring of the performance of hospitals and health systems.

### DEA model

The overall efficiency of any DMU has two major components, that is, technical and allocative efficiency. A DMU is considered to be technically efficient if it is able to produce maximum output from a given set of inputs. A DMU is allocatively efficient, if it is able to use the inputs in optimal proportions, given their respective costs. As the relevant data on costs of inputs were not available in this study, the allocative efficiency measures were not employed. We performed DEA with ‘input orientation’ considering the limited control of district hospitals over their outputs. Our study addresses the question: by how much can input quantities be proportionally saved without changing the output quantities produced?

The TE comprises pure technical and scale efficiency (SE) components. The SE puts a direct impact on the overall efficiency of the DMU. The increased scale of operations of a DMU results in economies or diseconomies of scale. In this context, the choice of assumption of variable returns to scale (VRS) or constant returns to scale (CRS) in estimating a DEA model becomes of critical importance. The CRS assumption focuses on productivity regardless of the scale of operations. Whereas, in the VRS assumption, interest is on the extent to which the scale of operation affects productivity. The VRS assumption is also preferred in the cases where all DMUs under analysis are not considered to be operating at an optimum scale. We carried out our analysis with the VRS assumption.

Returns to scale tell us how outputs respond in the long run to changes in the scale/size (inputs) of the hospital. The inappropriate size of a DMU might result in scale inefficiency, which can be further divided into two forms: decreasing returns to scale (DRS) and increasing returns to scale (IRS). The DRS denotes that the size of the DMU is very large for the volume of its operations (output increases by a smaller proportion than each of the inputs). However, a DMU exhibiting IRS is very small for its volume of activities and operations (output may increase by a larger proportion than each of the inputs). A scale-efficient DMU operates under CRS (5, 33, 40).

### Study variables

It is very important to select input and output variables in studies applying DEA. Hospitals turn inputs into outputs (health services) in the production process. The inputs are divided into three broad categories: labor (human resources), materials (drugs), and capital (buildings and equipment). It is widely acknowledged that the ultimate output in the production process of health facilities is improvement in population health. However, due to the measurement complexities and the availability of data for this type of analysis, it becomes difficult to assess the improvements in population health attributable to healthcare. Therefore, intermediate outputs are generally used as a preferred choice (6, 7, 33, 38, 40).

In modeling the health service production, we used three input and eight output variables in our study. The input variables for each district hospital were: (1) number of doctors (specialists and primary care physicians); (2) number of nurses; and (3) number of beds. The number of beds variable was included as a proxy indicator for capital inputs. The output variables were: (1) number of women with three completed antenatal checkups; (2) number of deliveries; (3) number of cesarean-section deliveries; (4) number of women receiving post-natal care within 48 hours of delivery (PNCs); (5) number of medical terminations of pregnancy (MTPs); (6) number of male and female sterilizations; (7) number of inpatient (IPD) admissions; and (8) number of outpatient (OPD) consultations.

The selection of the variables for this study was guided by a review of the literature on the hospital efficiency assessment using DEA, the availability of data, and our interest in maternal health services. The availability of data on various indicators was limited in the hospitals in Madhya Pradesh and, due to this constraint, we had to restrict our analysis to the above-mentioned input and output variables. Even we could not get data for the selected variables from 10 out of a total of 50 district hospitals; therefore, we could only include 40 district hospitals in our study. The data included in the study were fairly reliable as we conducted checks and found that they were of good quality. We conducted a data validation exercise in four randomly selected district hospitals (10% of the total sample) to check the accuracy and determine the reliability of the data. We also cross checked the data from the health management information system (HMIS), district reports, reporting formats, and hospital registers and no major inconsistencies were observed. To consider the broad range of services provided by the district hospitals, in addition to maternal healthcare services, the number of inpatient admissions and outpatient consultations were included as output variables.

### Data collection

We collected the data from the HMIS and other records of the Department of Public Health and Family Welfare, Government of Madhya Pradesh. Initially, all 50 public district hospitals in the state were planned to be included but data from 10 district hospitals on all variables could not be obtained. Data from 40 district hospitals from January to December 2010 were used.

### Analysis

First, descriptive statistics of all input and output variables were calculated by using Stata 11 software (Stata Corp. Inc., TX, USA). The mean, standard deviation (SD), minimum and maximum values of all input and output variables are presented. Subsequently, the TE scores were computed using the DEA Programme, version 2.1 (DEAP 2.1) developed by Tim Coelli (41). To be able to select a fewer number of variables for inclusion in the study, we conducted a correlation analysis among the output variables. The results of this analysis showed that, with a Spearman correlation coefficient greater than 0.7, none of the variables were associated.

To test the robustness of the DEA results regarding outlier district hospitals, Jack-knifing analysis was carried out. In this analysis, the efficient hospitals are removed one at a time and efficiency scores are recalculated. The efficiency rankings from the model prior to deleting any efficient DMUs and the new models, having removed each of the efficient DMUs, are then compared by using Spearman rank correlation coefficients. If the results are varied and not correlated, it means that efficient DMUs are influential. A value of 0 implies that there is no correlation between the rankings. A value of 1 (or −1) indicates that the rankings are exactly the same (or reverse), implying no influence of outliers on DMU efficiency (7, 8, 41–43). The results of this analysis in our study revealed no influence of outliers on district hospital efficiency.

## Ethics

Ethical approval for the study proposal was obtained from the Ethics Committee of the Bhopal Regional Technical Centre of the Family Planning Association of India and ethical standards were followed at all stages of this research.

## Results

This study used DEA to assess the TE of 40 district hospitals in the public sector from Madhya Pradesh. Table 2 presents the descriptive statistics of the variables of interest.

**Table 2:** Descriptive statistics of input and output variables, public district hospitals of Madhya Pradesh (January–December 2010)

Variable	Definition	Mean	Standard deviation	Minimum value	Maximum value
Inputs					
X1	# Doctors (specialists and primary care physicians)	29.17	12.68	12	75
X2	# Nurses	47.42	34	4	145
X3	# Beds	216.82	149.34	30	700
Outputs					
Y1	# Women with completed three antenatal checkups	3410.25	2451.99	531	11,019
Y2	# Deliveries	5828.25	2544.16	1,239	12,550
Y3	# C-section deliveries	493.97	555.29	1	2,454
Y4	# Women receiving post-natal care within 48 hours after delivery	5007.62	2703.58	1,094	12,505
Y5	# Medical termination of pregnancies	181.67	169.20	5	750
Y6	# Male and female sterilizations	1063.37	865.28	58	3,300
Y7	# Inpatient admissions	21594.6	19632.63	2,571	108,932
Y8	# Outpatient consultations	131089.5	116339.8	18,542	667,220

The VRS model technical and SE scores and returns to scale characteristics for individual district hospitals are given in Table 3. The mean scores of pure TE and SE of the district hospitals were 0.90 (SD=0.14) and 0.88 (SD=0.15), respectively. Of the total district hospitals included in the study, 20 (50%) were technically efficient constituting the ‘best practice frontier’. The other half was technically inefficient, with an average TE score of 0.79 (SD=0.12). This finding implies that these 20 inefficient district hospitals could potentially reduce their current input endowment by 21% while leaving their output levels unchanged. In other words, these 20 technically inefficient district hospitals could, on average, produce 21% more outputs by utilizing the current levels of inputs.

**Table 3:** Technical and scale efficiency scores and returns to scale characteristics of each public district hospital, Madhya Pradesh (January–December 2010)

District hospital	Technical efficiency score	Scale efficiency score	Type of scale inefficiency
Anuppur	0.786	0.384	Increasing return to scale (IRS)
Ashoknagar	1	0.913	IRS
Balaghat	1	1	–
Betul	0.765	0.895	IRS
Bhind	1	1	–
Bhopal	1	0.865	Decreasing return to scale (DRS)
Chhatarpur	0.874	0.961	IRS
Chhindwara	1	0.923	DRS
Damoh	1	1	–
Dewas	1	1	–
Dhar	0.602	0.659	IRS
Guna	1	1	–
Gwalior	1	1	–
Harda	0.884	0.881	IRS
Hoshangabad	1	1	–
Indore	0.666	0.678	IRS
Jabalpur	1	1	–
Jhabua	0.640	0.520	IRS
Katni	0.911	0.882	IRS
Khargone	0.769	0.989	IRS
Mandla	0.550	0.681	IRS
Mandsaur	0.890	0.931	IRS
Morena	1	1	–
Narsingpur	0.765	0.745	IRS
Panna	1	0.853	IRS
Raisen	0.655	0.548	IRS
Ratlam	0.812	0.948	IRS
Sagar	1	1	–
Satna	0.794	0.971	IRS
Sehore	0.751	0.960	IRS
Seoni	1	1	–
Shahdol	0.921	0.815	IRS
Shajapur	0.999	0.868	IRS
Sheopur	1	1	–
Shivpuri	1	1	–
Sidhi	1	1	–
Tikamgarh	0.935	0.871	IRS
Ujjain	1	0.688	DRS
Umaria	1	0.815	IRS
Vidisha	0.834	0.919	IRS

Fourteen (35%) district hospitals had an SE of 100%, implying thereby that they had the most productive scale size (MPSS) for that particular input–output mix. The remaining 26 (65%) hospitals were found to be scale inefficient, manifesting a mean SE score of 81% (SD=0.16). This implies that, on average, the scale-inefficient district hospitals could reduce their input size by 19% without affecting their current output levels.

Out of 26 scale-inefficient district hospitals, 23 (88.5%) manifested IRS and the remaining 3 (11.5%) revealed DRS. These findings reveal that 88.5% scale-inefficient district hospitals in Madhya Pradesh are too small for their operations and to operate at their MPSS, they need to expand their scale of operation. However, 11.5% of the inefficient district hospitals in the state need to scale down their operations for achieving the CRS.

Table 4 presents the total output increases and/or input reductions required for making the inefficient district hospitals efficient. The results show that, to become efficient, the inefficient district hospitals combined would need to reduce the number of doctors by 22%, number of nursing staff by 27%, and number of beds by 51.82% keeping the current output levels constant. Alternatively, the inefficient hospitals could become efficient by increasing the number of cases of women who had three complete antenatal check-ups by 30%, deliveries by 23%, C-section deliveries by 9%, PNCs by 42%, MTPs by 48%, sterilizations by 40%, IPD admissions by 12%, and OPD consultations by 22% with the current inputs.

**Table 4:** Total output (input) increases (reductions) needed to make inefficient public district hospitals efficient

Variables	Original value	Projection	Difference (%)
Outputs			
# Women with completed three antenatal checkups	48,396	63,001	30
# Deliveries	94,460	116,141	23
# C-section deliveries	7,600	8,292	9
# Women receiving post-natal care within 48 hours after delivery	77,090	109,348	42
# Medical termination of pregnancies	2,848	4,209	48
# Male and female sterilizations	15,892	22,207	40
# Inpatient admissions	382,546	427,489	12
# Outpatient consultations	2,004,332	2,442,283	22
Inputs			
# Doctors (specialists and primary care physicians)	527	412	−22
# Nurses	940	686	−27
# Beds	4,449	2,144	−51.82

## Discussion

This study is the first attempt at evaluating the technical efficiencies of district hospitals in Madhya Pradesh by using the DEA methodology. Though the Department of Public Health and Family Welfare of the state has significantly improved its HMIS in recent years, the study showed a considerable scope for further improvement in the HMIS as relevant data for 10 (20%) hospitals were missing.

The average pure TE score of 0.90 shows that the district hospitals included in the study can produce the same amount of outputs by saving 10% inputs. This implies that the input savings could be utilized to provide healthcare services to more people through CHCs situated in rural poor areas where these services are required. This could significantly contribute toward ensuring equitable availability of maternal healthcare services in the state. The results of this study showed that 50% of district hospitals are operating at less than optimal level and 11 of them obtained efficiency scores below 0.8. This finding implies that the inefficient hospitals could significantly improve their efficiency by better resource management. A positive point to note is that the TE scores of around half of the inefficient hospitals were quite high (above 0.8). The factors influencing efficiency of district hospitals should be identified and appropriately addressed. This may be achieved by conducting regression analysis of the environmental factors associated with these district hospitals using a Tobit analysis (8, 33) or by exploring these factors through qualitative research (44).

The finding of 50% district hospitals operating with technical inefficiency is similar to a study conducted in Gujarat state of India (17), whereas, a study conducted in Tamilnadu state found that 72% of the district hospitals were operating as technically inefficient during the year 2004–2005 (18). Another study conducted in Tamilnadu (19) revealed that 34.5, 41.3, 62, 55.2, and 51.7% of district hospitals were operating as technically inefficient during the years 2002–2003, 03–04, 04–05, 05–06, and 06–07, respectively. The differential percentage of inefficient district hospitals in our study and the Tamilnadu studies may be due to the different inputs–outputs, huge differences in socio-economic conditions of the states, and also the different periods of conducting the studies.

The study has also quantified the output (input) increases (reductions) required for making inefficient district hospitals efficient. The results of this analysis presented in Table 4 indicated a significant scope of input saving or increasing outputs of the inefficient hospitals. It would be important for these hospitals to ensure efficient utilization of the available resources through critical monitoring and improved management.

Although the aim of the study was not to establish a relationship between hospital efficiency and improved maternal health and obstetric outcomes, one would expect that increasing the TE (better use of existing resources) would lead to an increase in service coverage improving ultimately the health outcomes.

### Limitations of the study

This study has some limitations that need to be taken into account when interpreting the results. First, the output indicators were selected to represent the broad range of functions of the district hospitals while focusing on maternal health services. The reason behind is that this study is part of a larger research focusing on maternal health in Madhya Pradesh. The reasons for matching general inputs with partly general, partly specific outcomes in one analysis were: (1) information quality and availability from the hospitals; and (2) given the general inputs, to get a general perspective of the outputs, since the inputs used (doctors, nurses) work for providing services on maternal health as well as other services. Second, we are aware that the inclusion of more or different output indicators and the selection of other output–input mix in the study might have influenced the results. Third, the input and output data were collected for only 1 year which did not allow us to analyze and observe efficiency scores of district hospitals over the years. Fourth, the information on input costs could not be collected. Therefore, it was not possible to estimate the allocative efficiency.

## Conclusions

The findings of our study have significant policy implications for strengthening the healthcare delivery in the state. The results showed that 50% of district hospitals were operating as technically inefficient hospitals. Decision makers and administrators in the state should identify the causes of the observed inefficiencies and take appropriate measures to increase efficiency. Considering the poor health indicators of the state and scarcity of resources, ensuring efficient functioning of these hospitals will be of immense public health importance. The findings of this study are based on the particular input–output mix; therefore, the policy implications related to these findings should be considered within this perspective.

## References

[1] Planning Commission Government of India (2011). Faster, sustainable and more inclusive growth: an approach to twelfth five year plan (draft).

[2] Kirigia J, Sambo L, Scheel H (2001). Technical efficiency of public clinics in Kwa-Zulu Natal province of South Africa. East Afr Med J.

[3] Kirigia J, Emrouznejad A, Sambo L, Munguti N, Liambila W (2004). Using data envelopment analysis to measure the technical efficiency of public health centers in Kenya. J Med Syst.

[4] Kirigia J, Emrouznejad A, Gama Vaz R, Bastiene H, Padayachy J (2008). A comparative assessment of performance and productivity of health centres in Seychelles. Int J Prod Perform Manag.

[5] Akazili J, Adjuik M, Jehu-Appiah C, Zere E (2008). Using data envelopment analysis to measure the extent of technical efficiency of public health centres in Ghana. BMC Int Health Hum Rights.

[6] Osei D, d'Almeida S, George M, Kirigia J, Mensah A, Kainyu L (2005). Technical efficiency of public district hospitals and health centres in Ghana: a pilot study. Cost Eff Resour Alloc.

[7] Zere E, Mbeeli T, Shangula K, Mandlhate C, Mutirua K, Tjivambi B (2006). Technical efficiency of district hospitals: evidence from Namibia using data envelopment analysis. Cost Eff Resour Alloc.

[8] Guven-Uslu P, Linh P (2008). Effects of changes in public policy on efficiency and productivity of general hospitals in Vietnam.

[9] Renner A, Kirigia J, Zere E, Barry S, Kirigia D, Kamara C (2005). Technical efficiency of peripheral health units in Pujehun district of Sierra Leone: a DEA application. BMC Health Serv Res.

[10] Borden JP (1988). An assessment of the impact of Diagnosis-Related Group (DRG)-based reimbursement on the technical efficiency of New Jersey hospitals using data envelopment analysis. J Account Publ Pol.

[11] Lopez-Valcarcel BG, Perez PB (1996). Changes in the efficiency of Spanish public hospitals after the introduction of program-contracts. Invest Econ.

[12] Chang HH (1998). Determinants of hospital efficiency: the case of central government-owned hospitals in Taiwan. Int J Manag Sci.

[13] Chern JY, Wan TTH (2000). The impact of the prospective payment system on the technical efficiency of hospitals. J Med Syst.

[14] Chu HL, Liu SZ, Romeis JC (2004). Does capitated contracting improve efficiency? Evidence from California hospitals. Health Care Manage Rev.

[15] Biørn E, Hagen TP, Iversen T, Magnussen J (2003). The effect of activity-based financing on hospital efficiency: a panel data analysis of DEA efficiency scores 1992–2000. Health Care Manag Sci.

[16] Ersoy E, Kavuncubasi S, Ozcan YA, Harris JM (1997). Technical efficiency of Turkish hospitals: DEA approach. J Med Syst.

[17] Bhat R, Verma BB, Reuben E (2001). Hospital efficiency: an empirical analysis of district hospitals and grant in aid hospitals in Gujarat. J Health Manag.

[18] Dash U, Vaishnavi S, Muraleedharan V, Acharya D (2007). Benchmarking the performance of public hospitals in Tamilnadu: an application of data envelopment analysis. J Health Manag.

[19] Dash U (2009). Evaluating the comparative performance of district head quarters hospitals, 2002–07: a non-parametric Malmquist approach.

[20] Shetty U, Pakkala TPM (2010). Technical efficiencies of healthcare system in major states of India: an application of NP-RDM of DEA formulation. J Health Manag.

[21] Hollingsworth B, Dawson PJ, Maniadakis N (1999). Efficiency measurement of health care: a review of non-parametric methods and applications. Health Care Manag Sci.

[22] Hollingsworth B (2003). Non-parametric and parametric applications measuring efficiency in health care. Health Care Manag Sci.

[23] O'Neil L, Rauner M, Heidenberger K, Kraus M (2008). A cross-national comparison and taxonomy of DEA-based hospital efficiency studies. Soc Econ Plann Sci.

[24] Worthington AC (2004). Frontier efficiency measurement in health care: a review of empirical techniques and selected applications. Med Care Res Rev.

[25] Registrar General of India (2011). Sample registration system-bulletin on maternal mortality in India 2007–2009.

[26] Department of Public Health and Family Welfare—Government of Madhya Pradesh (2010). National rural health mission-state project implementation plan.

[27] Registrar General of India (2011). Census of India, 2011—provisional population totals, Madhya Pradesh.

[28] National Sample Survey Organisation (2007). Consumer expenditure, employment–unemployment survey 61st round, 2004–05.

[29] Ministry of Health & Family Welfare—Government of India (2011). Rural health care system in India.

[30] Ministry of Health & Family Welfare—Government of India (2011). Bulletin on rural health statistics in India 2010.

[31] Department of Public Health & Family Welfare—Government of Madhya Pradesh (2010). Health institutions in Madhya Pradesh—2010.

[32] Skinner J (1994). What do stochastic frontier cost functions tell us about inefficiency?. J Health Econ.

[33] Jacobs R, Smith P, Street A (2006). Measuring efficiency in health care: analytic techniques and health policy.

[34] Valdmanis V, Kumarnayake L, Lertiendumrong J (2004). Capacity in Thai public hospitals and the production of care for poor and nonpoor patients. Health Serv Res.

[35] Charnes A, Cooper WW, Rhodes E (1978). Measuring of efficiency of decision making units. Eur J Oper Res.

[36] Banker RD, Charnes A, Cooper WW (1984). Some models for estimating technical and scale inefficiencies in data envelopment analysis. Manag Sci.

[37] Farrell MJ (1957). The measurement of productive efficiency. J Roy Stat Soc A.

[38] Coelli T, Rao D, Battese G (2005). An introduction to efficiency and productivity analysis.

[39] Grosskopf S, Valdmanis V (1987). Measuring hospital performance: a non-parametric approach. J Health Econ.

[40] Sebastian M, Lemma H (2010). Efficiency of the health extension programme in Tigray. Ethiopia: a data envelopment analysism BMC Int Health Hum Rights.

[41] Coelli T (1996). A guide to DEAP version 2.1: a data envelopment analysis (computer) program.

[42] Efron B (1982). The jackknife, the bootstrap and other resampling plans.

[43] Magnussen J (1996). Efficiency measurement and the operationalization of hospital production. Health Serv Res.

[44] Afzali HHA, Moss JR, Mahmood MA (2011). Exploring health professionals’ perspectives on factors affecting Iranian hospital efficiency and suggestions for improvement. Int J Health Plann Manage.

